# Liver transplantation versus resection for patients with combined hepatocellular cholangiocarcinoma: A retrospective cohort study

**DOI:** 10.1016/j.heliyon.2023.e20945

**Published:** 2023-10-13

**Authors:** Shizheng Mi, Ziqi Hou, Guoteng Qiu, Zhaoxing Jin, Qingyun Xie, Jiwei Huang

**Affiliations:** Department of Liver Surgery and Liver Transplantation Center, West China Hospital, Sichuan University, Chengdu, China

**Keywords:** Liver transplantation, Hepatectomy, Combined hepatocellular cholangiocarcinoma, Overall survival, Cancer-specific survival

## Abstract

**Background:**

Combined hepatocellular cholangiocarcinoma (CHC) is a rare primary liver cancer, and whether liver transplantation should be implemented among CHC patients is still controversial. We intend to conduct a retrospective cohort study based on the Surveillance, Epidemiology, and End Results (SEER) database to investigate the prognosis of liver transplantation *vs.* liver resection among CHC patients.

**Methods:**

Patients diagnosed with CHC (ICD-O-3:8180/3) and treated with transplantation or hepatectomy were extracted from the SEER database (2000–2018). We utilized Propensity Score Matching to control confounding bias. Kaplan-Meier curve was used for survival analysis, and Cox regression was used to find independent factors associated with prognosis.

**Results:**

We identified 123 (transplantation: 49; resection: 74) patients with CHC who were treated between 2004 and 2015. In the entire cohort, survival analysis demonstrated transplantation group was associated with better overall survival and cancer-specific survival (log-rank p = 0.004 and p = 0.003, respectively). In addition, liver transplantation still conferred better overall and cancer-specific survival than liver resection after Propensity Score Matching (log-rank p = 0.024 and p = 0.048, respectively). However, this advantage didn't appear in the subgroup, regardless of whether the tumor size was greater than 3 cm or not. (≤3 cm: OS log-rank p = 0.230, CSS log-rank p = 0.370; >3 cm: OS log-rank p = 0.110, CSS log-rank p = 0.084). Multivariate analysis validated the finding that liver transplantation was a protective factor for overall survival (HR = 0.55 [0.31–0.95], p = 0.032).

**Conclusions:**

Liver transplantation may be an option in individuals with CHC and should be taken into consideration due to its advantages in terms of overall survival and cancer-specific survival. However, a sizable sample is required for future studies to determine which subset of CHC patients may benefit more from liver transplantation.

## 1 Introduction

Primary liver cancer (PLC) is the seventh most common cancer and the fourth leading cause of cancer-related death worldwide [1]. It has also brought a great burden to China's health system, with its mortality ranking second only to lung cancer [2]. The two major malignancies, hepatocellular carcinoma (HCC) and intrahepatic cholangiocarcinoma (iCCA) are by far the most prevalent and represent two distinct spectrums of primary liver cancer [3,4]. Nevertheless, a heterogeneous tumor type derived from the differentiation of hepatocytes and cholangiocytes was observed 100 years ago but only in recent years has a uniform terminology, termed “combined (or mixed) hepatocellular bile ducts” cell carcinoma (cHCC-CCA)” or simplified to “Combined hepatocellular cholangiocarcinoma (CHC)” [5]. This type of PLC is quite uncommon, with an incidence rate of 0.05 per 100,000 people per year in western populations [6]. In eastern and western populations, the prevalence of CHC in PLC ranges from 3 % to 5 %, and the extremely low incidence results in slow progress in relevant research [7,8].

Allen et al. [9] first provided the following initial description of the CHC categorization in 1949: (Ⅰ). Two separate nodules of hepatocellular and bile duct cancer make up the CHC nodule. (Ⅱ) Adjacent masses with different characters, which can intermingle with each other as they grow. (Ⅲ). Real mixed tumors are assumed to have a common origin. Then in 1985, Goodman et al. renewed the histopathological categorization of CHC, classifying it into three types: A: “collision tumor”, B: “transitional tumor” and C: “fibrolamellar tumor” [10]. According to the WHO Classification of Tumors, 4th Edition, Digestive System Tumors, it is recommended to divide CHC into the following two categories [1]: Classical CHC, hepatocyte and cholangiocyte differentiation present within the same tumor [2]. Stem cells-CHC, including “intermediate cell”, “typical”, and “cholangiolocellular” subtype [11]. Nevertheless, the diagnostic category “stem cells-CHC” has been removed in the 5th Edition. The cholangiolocellular subtype is no longer considered part of CHC but is a subtype of small ductal intrahepatic cholangiocarcinoma [12]. Besides “classical CHC”, another type is “intermediate cell carcinoma”, which means that the cancer cells have a morphology between liver cells and bile duct cells and express tumor markers of both hepatocellular carcinoma (HCC) and cholangiocarcinoma (CCA). Despite the WHO's ongoing efforts to improve the criteria for CHC, there are few is little data on the effectiveness of biopsy in diagnosing CHC. According to estimates, a significant portion of CHC cases is currently misdiagnosed as HCC [13].

Due to the tiny population and changing classification over time, the treatment and prognosis of CHC are still uncertain and controversial. CHC has similar clinical features as HCC, including sex, HBV infection, AFP and PIVKA-Ⅱ, but more frequent lymph node metastasis and vascular invasion compared with HCC [14]. As a result, the long-term survival of CHC is between that of HCC and intrahepatic cholangiocarcinoma (iCCA) and is generally comparable to iCCA. In 2014, Garancini et al. [15] confirmed that active surgical intervention can significantly improve the prognosis of CHC. The disease-specific survival of patients who received liver transplantation (LT) or liver resection (LR) was higher than that of patients who underwent radiofrequency ablation or non-invasive therapies.

So far, the status of liver transplantation in CHC remains much debated [16]. To determine the best course of treatment for CHC patients, the Surveillance, Epidemiology and End Results (SEER) database was harnessed to make a comparison between the prognostic outcomes of LT *vs.* LR among CHC patients.

## 2 Materials and methods

**Fig. 1:**
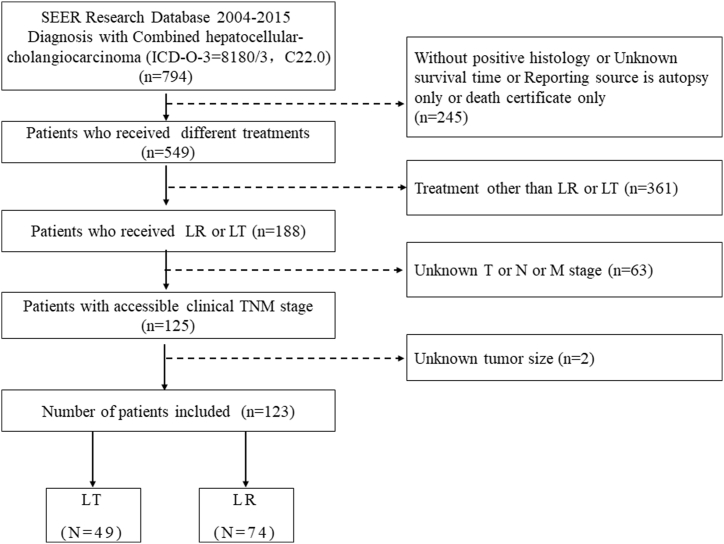
Flowchart of patients screening.

For this study, data from SEER 18 registries from 2004 to 2015 were gathered [17]. Firstly, the International Classification of Diseases for Oncology, 3rd Edition (ICD-O-3) histologic type code 8180 and site code C22.0 were selected to ensure that all included patients were diagnosed with CHC (N = 794). In addition, patients without positive histology or available survival time were excluded. Moreover, reporting sources of only autopsies or death certificates were not eligible for this study. The remaining 549 patients were all treated between 2004 and 2015 and the flow diagram of patients screening is presented in Fig. 1. We excluded 361 patients receiving treatment other than LT or LR and 63 patients with unknown T, N, or M stage and 2 patients with unknown tumor size. In the end, 123 individuals were enrolled in this trial. (Transplant group: 49 cases; Hepatectomy group: 74 cases).

The demographic variables were age, sex, grade of tumor differentiation, race, tumor size, treatment, AJCC TNM stage, regional lymph node status, and extent of regional lymph node surgery. The age of patients was divided into two groups: ≥65 & <65. White, black, and other races were listed as the race. The tumor differentiation was classified into three groups: poor differentiation-undifferentiation (Grade Ⅲ-Ⅳ); well-moderate differentiation (Grade Ⅰ-Ⅱ); and unknown. The extent of regional lymph node surgery refers to the removal, biopsy, aspiration, or absence of these operations. Correspondingly, the regional lymph nodes that underwent the above operations were recorded as “positive” or “negative”, and those that lacked the above procedures were recorded as “not examined”. The American Joint Committee on Cancer (AJCC) stage was categorized as AJCC Ⅰ-Ⅱ and AJCC Ⅲ-Ⅳ according to the 6th cancer staging systems. The treatment code in the SEER database for CHC includes: LR: 20–26, 36–38, 50, 51, 60, LT:61,75.

Because all patients’ information in the SEER database has been deidentified and is publicly accessible, the research involving the anonymous data from that database was exempt from medical ethics review and did not require informed consent.

The main outcomes were taken to be overall survival (OS) and cancer-specific survival (CSS), which are frequently used to assess prognostic outcomes. The period between the day of diagnosis and any sort of death was defined as OS. The time until death attributed to CHC was referred to as CSS.

R software (version 4.2.2) was used for statistical analysis and graph creation in this work. Mean ± SD and Counts are the presentation formats for continuous and categorical variables, respectively. The former is examined by *t*-test, and the latter is examined by Χ^2^ or Fisher exact test. Using the “MatchIt” package, we performed Propensity Score Matching to balance the covariates between the two groups [18]. Consistent with the explanation of this package, logistic regression was constructed with the surgical approach as the “treatment variable” and the remaining variables as covariates, with the goal of creating a distance measure for matching. Subsequently, we set method = “nearest” to perform nearest neighbor matching at a 1:1 ratio. Finally, when the caliper value was set to 1.2, the matched cohort eliminated the impact of confounding bias and maintained the optimal sample size. Survival analysis was visualized by Kaplan-Meier curves and analyzed using the log-rank test. The factors that were independently related to survival were discovered using Cox regression. Considering that OS and CSS are time-to-event data, we used HR (Hazard Ratio) with 95%CI (Confidence interval) to show the relative risk of different groups.

The above steps are implemented through the following two packages, namely “survival” and “survminer” [19,20]. Variables were screened using univariate Cox regression, and significant indicators were selected in multivariate Cox regression for further analysis. The aforementioned two procedures were used to confirm the independent factors associated with CHC. In this study, p-value <0.05 (two-sided) is a symbol of statistical significance.

## 3 Results

### 3.1 Characteristics of the entire group and the PSM group

Forty-nine (39.84 %) of the 123 CHC patients received LT, and seventy-four (60.16 %) received LR in the entire cohort. The patients’ characteristics are shown in Table 1. Patients undergoing LR were older, and more often male. The mean tumor size of CHC patients was 62.58 ± 36.69 in the LR group and 25.71 ± 13.39 in the LT group. Cases with LR had a larger tumor size than that of LT. It is obvious that patients with AJCC stage III-IV and tumor differentiation grade Ⅲ-Ⅳ were more common in the LR group. After 1:1 PSM, a total of 34 pairs were comprised in this study. In the matching cohort, all eight variables were well-balanced (Table 2). There was no statistical significance in each item of the two groups.

**Table 1:** Baseline of the entire cohort.

	LR (n = 74)	LT（n = 49）	P value
Age (years, <65/≥65)	42/32	42/7	<0.001^a^
Sex (female/male)	28/46	10/39	0.041^a^
Race（white/black/other）	50/7/17	41/4/4	0.093
Tumor size (mm)	62.58 ± 36.69	25.71 ± 13.39	<0.001^a^
Tumor differentiation (I-II/III-IV/unknown)	25/37/12	28/9/12	0.002^a^
AJCC stage 6th (I-II/III-IV)	51/23	44/5	0.007^a^
Regional lymph nodes status (negative/positive/Not examined)	18/5/51	17/2/30	0.467
Removal or biopsy or aspiration of regional Lymph Nodes (no/yes)	52/22	33/16	0.731

a Statistically significant.

**Table 2:** Baseline of the matched cohort.

	LR (n = 34)	LT（n = 34）	P value
Age (years, <65/≥65)	22/12	27/7	0.177
Sex (female/male)	9/25	8/26	0.779
Race（white/black/other）	23/3/8	26/4/4	0.466
Tumor size (mm)	36.18 ± 17.51	28.79 ± 13.42	0.056
Tumor differentiation (I-II/III-IV/unknown)	15/10/9	16/9/9	0.958
AJCC stage 6th (I-II/III-IV)	29/5	31/3	0.707
Regional lymph nodes status (negative/positive/Not examined)	7/2/25	13/1/20	0.376
Removal or biopsy or aspiration of regional Lymph Nodes (no/yes)	25/9	23/11	0.595

### 3.2 Survival analysis of the whole cohort and the matched cohort

#### 3.2.1 Entire cohort

**Fig. 2:**
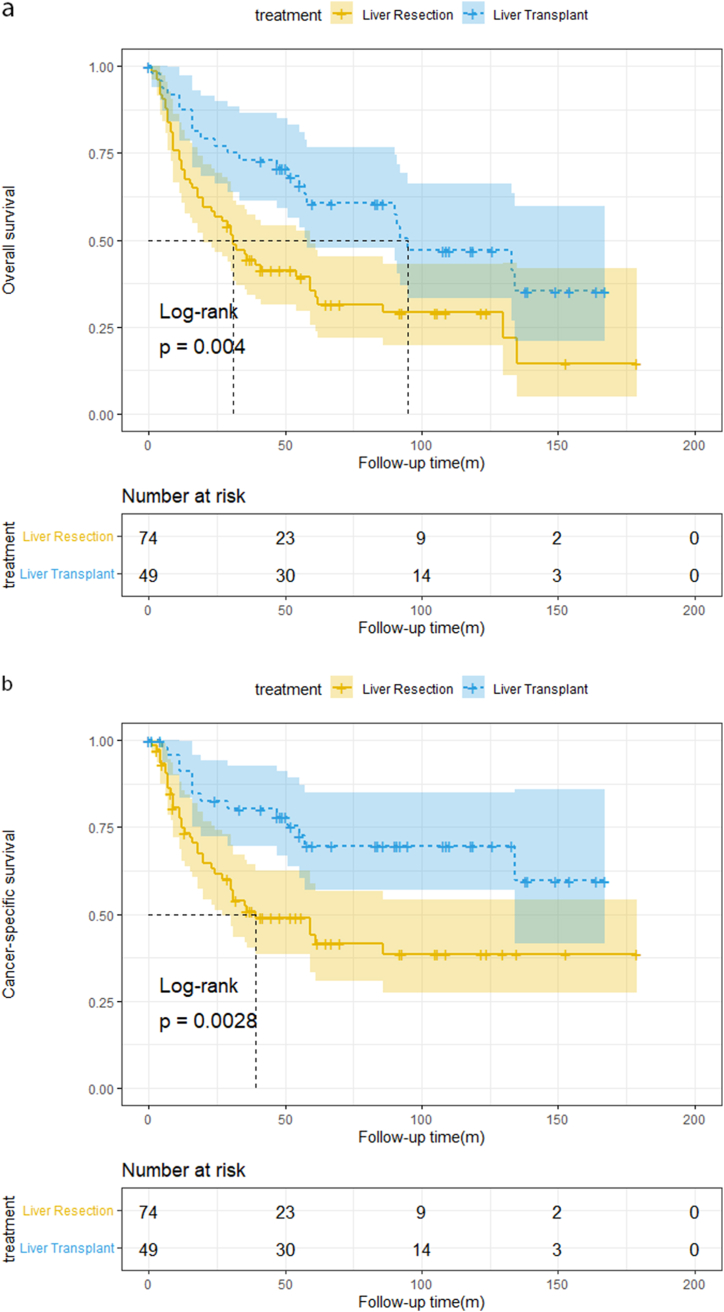

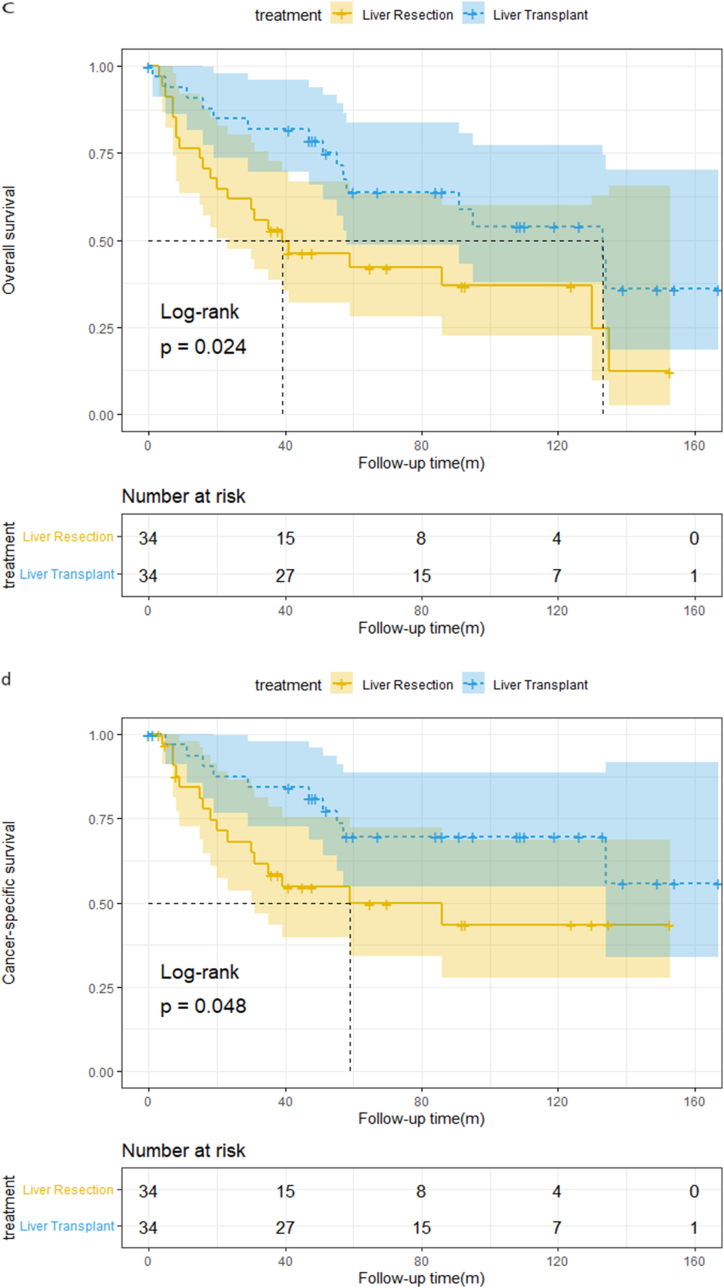
a)OS curves for 123 CHC patients Fig. 2b: CSS curves for 123 CHC patients Fig. 2c: OS curves for 68 CHC patients Fig. 2d: CSS curves for 68 CHC patients.

In the entire cohort, the median OS was 30 and 92 months in the LR and LT groups, respectively, and the median CSS for the LR cohort was 36 months. When the final follow-up occurred, CSS in the LT group was still higher than 50 %. After the median follow-up of 41(0–179) months, the overall patient survival rate for those undergoing LR was 70.3 %, 44.4 %, and 34.5 % at one, three, and five years, respectively and the one-, three-, and five-year CSS was 74.8 %, 50.8 %, and 42.9 %, respectively. For patients undergoing LT, the one-, three-, and five-year OS was 86.2 %, 72.4 %, and 60.3 % respectively; the one-, three-, and five-year CSS was 90 %, 79.0 %, and 69.3 %, respectively. The OS and CSS curves for CHC patients are shown in Fig. 2a&2b and the log-rank test revealed that patients who received LT had superior OS and CSS than those who underwent LR (log-rank p = 0.004 and p = 0.003, respectively).

#### 3.2.2 Matched cohort

The median OS was 39 and 114 months in the LR and LT group, respectively, and the median CSS for the LR cohort was 59 months. When the final follow-up occurred, CSS in the LT group was still higher than 50 %. After the median follow-up of 48.5 (0–167) months, overall patient survival for those undergoing LR was 75 %, 52.1 %, and 42.1 % at one, three, and five years, respectively, and the one-, three-, and five-year CSS was 82.7 %, 58.4 %, and 49.8 %, respectively. For patients undergoing LT, the one-, three-, and five-year OS was 90.3 %, 80.6 %, and 63.5 %, respectively; the one-, three-, and five-year CSS was 93.2 %, 83.1 %, and 69.2 % respectively. The OS and CSS curves for CHC patients are shown in Fig. 2c and d and the log-rank test revealed that patients who received LT had superior OS and CSS than those who underwent LR (log-rank p = 0.024 and p = 0.048, respectively).

### 3.3 Subgroup analysis

A subset analysis based on tumor size was conducted to further control for disease-related confounding bias between the two groups. Focusing on multiple tumors with a diameter **≤**3 cm seems to be clinically important, as iCCA patients who meet Milan criteria also appear to have favorable post-LT outcomes [21]. Given that the biological features of CHC may be better than iCCA, CHC patients within Milan criteria may also benefit from transplantation. This raises the question that the tumor size cutoff maybe 3 cm.

#### 3.3.1 Analysis of patients with CHC ≤3 cm

**Fig. 3:**
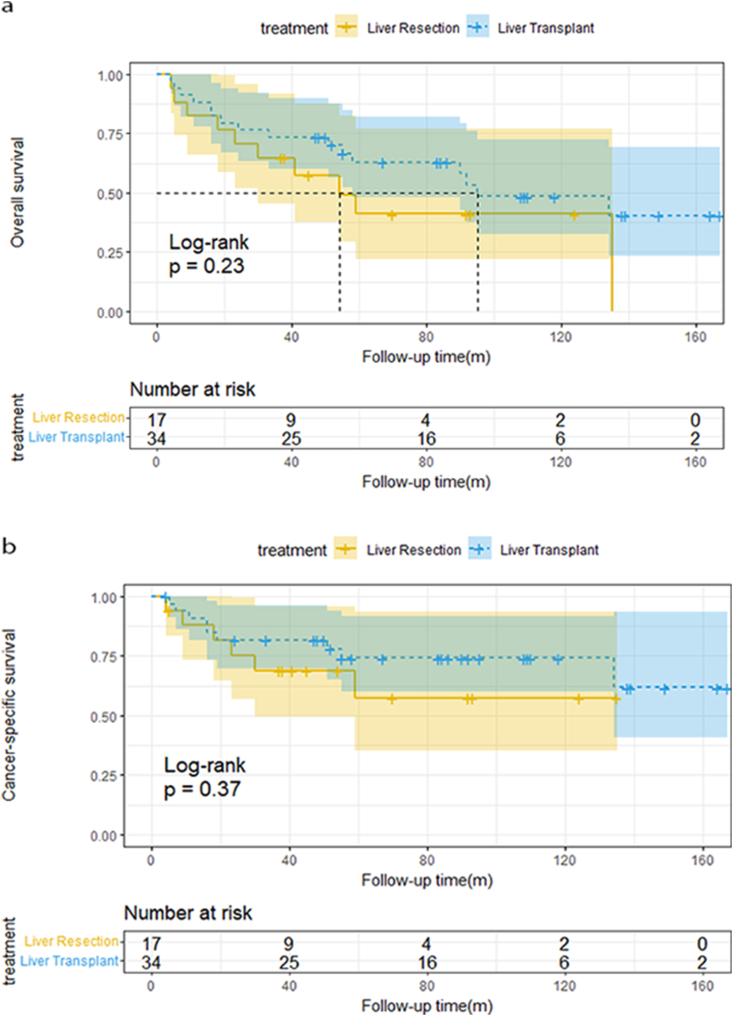

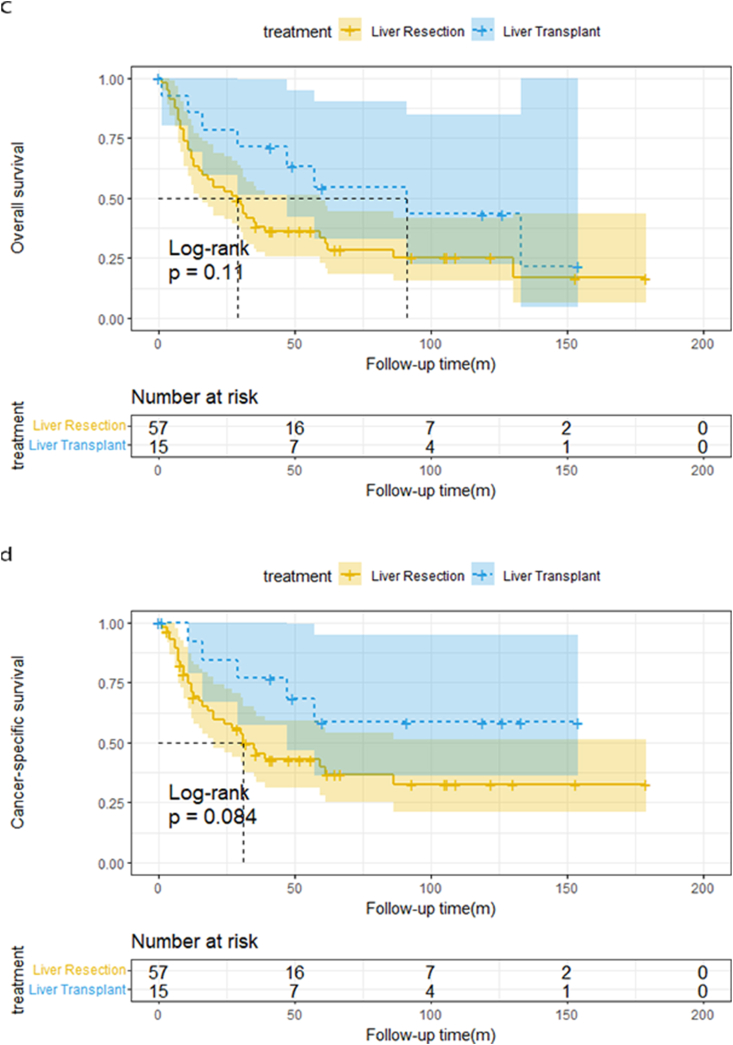
a)OS curves of CHC patients with tumor burden ≤3 cm Fig. 3b: CSS curves of CHC patients with tumor burden ≤3 cm Fig. 3c: OS curves of CHC patients with tumor burden >3 cm Fig. 3d: CSS curves of CHC patients with tumor burden >3 cm.

The tumor burden of 51 patients was no more than 3 cm; 17 of them underwent LR, while 34 of them underwent LT. After the median follow-up of 55(4–167) months, the median OS for LR and LT were 54 and 94 months, respectively, whereas the median CSS for LR and LT was not reached until the end of the follow-up. The one-year, three-year, and five-year OS of liver resection are 82.4 %, 58.0%, and 41.1 %, respectively. (Liver transplantation:88.2 %, 73.0 %, and 63 %, respectively) while one-year, three-year, and five-year CSS of liver resection are 85 %, 69 %, and 57.5 %, respectively. (Liver transplantation:90.9 %, 80 %, and 74.2 %, respectively). The log-rank test revealed that patients undergoing LT had a similar OS with LR (log-rank p = 0.23), and there was no significant difference between the two groups in terms of CSS (log-rank p = 0.37), as shown in Fig. 3a&3b.

#### 3.3.2 Analysis of patients with CHC >3 cm

Only 15 CHC patients with tumor burden over 3 cm received transplantation, the other 57 cases all underwent hepatectomy. Since multiple tumors>3 cm exceed Milan criteria, even for individuals with HCC, liver transplantation is not recommended as first-line treatment, and therefore, hepatic resection is preferred in these populations. After the median follow-up of 30.5(0–179) months, the median OS for LR was 27 months, while the median CSS for LR was 30 months. Overall patient survival for those undergoing LR was 66.7 %, 38.2 %, and 33.7 % at one, three, and five years, respectively, and the one-, three-, and five-year CSS was 71.1 %,45.2 %, and 39.9 %, respectively. Due to the small number of cases in the LT group, we didn't report a median survival time. Among patients with tumor size>3 cm, there was no significant discrepancy in long-term survival between hepatic resection and transplant. (OS: log-rank p = 0.11, CSS: log-rank p = 0.084).

### 3.4 Regression analysis

After survival analysis, independent factors affecting the prognosis of CHC patients were further explored. Univariate and multivariate analyses of the impact of patient demographics, tumor features, and clinical parameters on overall patient survival are shown in Table 3. Univariate Cox regression indicated that treatment (LT *vs.* LR), tumor size, and AJCC stage (Ⅲ-Ⅳ *vs.* Ⅰ-Ⅱ) were likely independent factors of OS. Therefore, we selected these variables in the multivariate regression model. Multivariate analysis, which is consistent with survival analysis, revealed that LT confers an advantage for overall patient survival in people with CHC. (HR = 0.55 [0.31–0.95], p = 0.032). In addition, patients with advanced AJCC stage had poorer OS (HR = 3.14 [1.72–5.13], p < 0.001). However, the OS of CHC patients is unaffected by tumor size. (HR = 1.00 [0.99–1.00], P = 0.345).

**Table 3:** Univariate and multivariate analysis of overall survival.

	Univariable model		Multivariable model	
	HR [95 % CI]	p	HR [95 % CI]	p
LT *vs.* LR	0.49 [0.30–0.81]	0.005^a^	0.55 [0.31–0.95]	0.032^a^
Age (years), >65 *vs.*<65	1.31 [0.81–2.10]	0.266		
Sex, male *vs.* female	0.89 [0.55–1.44]	0.628		
Race				
Black *vs.* White	0.94 [0.41–2.20]	0.893		
Other *vs.* White	1.04 [0.57–1.90]	0.900		
Tumor size, mm	1.01 [1.00–1.01]	0.016^a^	1.00 [0.99–1.00]	0.345
AJCC stage, Ⅲ-Ⅳ vs Ⅰ-Ⅱ	3.12 [1.90–5.11]	<0.001^a^	3.14 [1.72–5.73]	<0.001^a^
Regional LN status				
Positive *vs.* Negative	2.22 [0.89–5.53]	0.087		
Not examined *vs*. Negative	0.85 [0.51–1.43]	0.544		
Removal or biopsy or aspiration of regional LN, yes vs no	1.33 [0.81–2.17]	0.255		

a Statistically significant

Similarly, in the univariate analysis, treatment (LT or LR), tumor size, and AJCC stage (Ⅲ-Ⅳ *vs.* Ⅰ-Ⅱ) were also positive CSS-related factors (Table 4). In the univariate model, LT was related to better CSS (HR = 0.40 [0.22–0.75], p = 0.004) compared with LR. Bigger tumor size (HR = 1.01 [1.00–1.01], p = 0.002) and advanced AJCC stage (HR = 3.13 [1.81–5.70], p < 0.001) were significantly related to poorer CSS. After multivariate analysis, we found that only AJCC stage was an independent factor linked to CSS (HR = 2.58 [1.29–5.16], p = 0.007). Notably, LT was associated with a trend toward better CSS, but not statistically (HR = 0.51 [0.25–1.02], p = 0.057).

**Table 4:** Univariate and multivariate analysis of cancer-specific survival.

	Univariable model		Multivariable model	
	HR [95 % CI]	p	HR [95 % CI]	p
LT *vs.* LR	0.40 [0.22–0.75]	0.004^a^	0.51 [0.25–1.02]	0.057
Age (years), >65 *vs.* <65	0.96 [0.53–1.72]	0.882		
Sex, male *vs.* female	0.79 [0.45–1.37]	0.395		
Race				
Black *vs*. White	0.81 [0.29–2.26]	0.683		
Other *vs*. White	1.01 [0.49–2.09]	0.969		
Tumor size, mm	1.01 [1.00–1.01]	0.002^a^	1.00 [0.99–1.01]	0.909
AJCC stage, Ⅲ-Ⅳ *vs.* Ⅰ-Ⅱ	3.13 [1.81–5.70]	<0.001^a^	2.58 [1.29–5.16]	0.007^a^
Regional LN status				
Positive *vs.* Negative	1.58 [0.45–5.51]	0.476		
Not examined *vs*. Negative	0.98 [0.53–1.82]	0.951		
Removing or biopsy or aspiration of regional LN, yes *vs.* no	1.18 [0.65–2.12]	0.588		

a Statistically significant.

## 4 Discussion

Based on the SEER database, our current study reveals the advantage of liver transplantation in CHC. Survival analysis and Cox regression consistently demonstrated the benefit of liver transplantation on OS and CSS. Confounding bias was effectively corrected by propensity score matching, and in the matched cohort, the LT group outperformed the LR group in terms of both overall and cancer-specific survival. However, subset analysis demonstrated that liver transplantation didn't confer an advantage to those with tumor size ≤3 cm and there was no difference in OS and CSS between liver transplantation and liver resection among patients with tumor burden >3 cm. Additionally, Cox regression indicated that the AJCC stage and tumor size also had an impact on long-term survival.

The classification of CHC has changed many times over the past 40 years, leaving little room for research and a “grey area” of therapeutic options [13]. In 2014, Garancini et al. confirmed that liver transplantation or hepatectomy can significantly improve the prognosis compared with radiofrequency ablation or non-invasive treatment [15]. Consequently, surgical excision continues to be the cornerstone of therapy and the only well-recognized curative strategy, offering the patient greater survival benefits [22]. Yet, there is ongoing debate over the function of liver transplantation in CHC. It has been pointed out that the majority of CHC patients undergoing LT are considered a waste of organ resources and ought to be avoided [16]. This is because that transplantation for CHC provides a survival advantage comparable to hepatectomy but appears to be less beneficial than liver transplantation for HCC [23,24]. The majority of earlier investigations relied on public databases because of the low prevalence and challenging diagnosis of CHC. There is also a center reaching the same conclusion based on their database and considering CHC or cholangiocarcinoma as relative contraindications for LT [25]. In contrast, in 2021, a multicenter retrospective study including 2998 HCC patients and 208 CHC patients confirmed that CHC patients who received transplantation showed significantly better OS and DFS compared with resection, regardless of tumor burden [26]. Their findings are in line with those of our study, which contends that LT is preferable to resection for CHC patients due to its superior survival outcome. The study further pointed out that within the Milan criteria, there was no significant difference in the 5-year OS between LT for HCC and LT for CHC. However, in the entire cohort, LT for HCC was connected with better OS, and lower recurrence rates compared with LT for CHC [26]. These results suggest the possibility that CHC patients within Milan criteria could be candidates for liver transplantation. The study's conclusions apparently contradict previous studies based on SEER and UNOS databases [23,24]. From our perspective, the reasons may be attributed to two aspects: 1. Due to the retrospective harness of large databases, these studies was unable to distinguish outcomes stratified by clinical stage (e.g., Milan criteria) and was lack of certain data. 2. Baseline differences haven't been calibrated by statistical methods. (e.g., PSM).

Recent studies have likewise indicated positive outcomes for CHC patients receiving liver transplants. Lunsford et al. matched 12 patients with CHC with 36 patients with HCC, suggesting that low-grade, well-moderate differentiated CHC with favorable outcomes and low risk of recurrence should be permitted for LT [27]. Jaradat et al. conducted a multicenter analysis of 19 patients with CHC and concluded that individuals with early-stage and histological signs of both HCC and CCA within a single lesion should be given transplant consideration [28]. Facciuto et al. discovered that individuals within Milan criteria had a 78 % 5-year survival rate following transplantation in a cohort study of 32 patients with iCCA (half of them had CHC) [21]. De Martin et al. [29] conducted a subgroup analysis of tumor diameter and observed that cirrhosis iCCA/CHC patients who underwent liver transplants had considerable 5-year RFS regardless of tumor diameter. (5-year RFS of patients with iCCA/CHC <2 cm: 75 %; 5-year RFS of patients with iCCA/CHC >2 cm but ≤5 cm: 74 %) Moreover, among patients with tumor burden <2 cm, transplantation can significantly reduce tumor recurrence in comparison to liver resection. This study including 75 patients, in line with previous studies, was restricted by the limited sample size. Additionally, since the diagnosis was confirmed after the transplant, the dropout rate among CHC patients due to tumor progression couldn't be evaluated, which led to inevitable selection bias. What's more, the biometric heterogeneity of iCCA and CHC makes them unsuitable for analysis in a cohort, which is also a limitation of current studies. Hence, a relatively large-sample study is required to figure out whether patients with CHC are suitable for liver transplantation.

Based on the SEER database, only four studies—two of which have already been mentioned—have examined the role of liver transplantation in CHC [[15], [23]]. The conclusions of the remaining two studies were different [30,31]. Wang et al. hold the belief that patients undergoing LT had the same prognosis as those who underwent LR, and this was still the case when selecting patients with AJCC stages I and II [30]. However, Chen et al. suggested that LT can bring long-term survival advantages for patients with CHC, which is consistent with our results [31]. The limitation of the former is that local tumor destruction and radiofrequency ablation are also considered as surgical treatment, and there is no way to deal with confounding bias [30]. The limitation of the latter is that there is too much unknown information in the included patients, and the multivariate analysis does not include clinical stage, and regional lymph node status, which are of vital importance for the long-term survival or recurrence among CHC patients. And transform the tumor size from a continuous variable into a binary variable and set 2 cm as the cut-off value, which may affect the accuracy of the analysis results [31].

Our study has some limitations: Firstly, based on a large public database, it is inevitable that some patients' information is inaccessible. When it comes to tumor characteristics, only tumor size and tumor differentiation are available. However, due to the lack of these information, our model didn't include tumor biomarkers, microvascular invasion, and tumor number. As certain oncological parameters are associated with prognosis, future studies should include as many of these variables as possible. Secondly, consistent with the vast majority of studies, due to the retrospective cohort nature of our investigation, there may be selection bias. As mentioned above, almost all CHC patients were misdiagnosed with HCC at the first time, which hinders the implementation of prospective studies. Clear classification and more sensitive diagnostic methods are in desperate need for CHC patients. What's more, subgroup analysis didn't show that CHC patients within Milan criteria benefited more from liver transplantation, which may be attributed to insufficient sample size. Nonetheless, we maintain that our cohort still represents one of the largest cohorts in the literature. Finally, most of the included patients are in the early to intermediate stage (AJCC Ⅰ-Ⅱ) and some features of advanced tumors like macrovascular invasion, and portal vein tumor thrombus, are unknown. Furthermore, the long-term survival of LT for HCC patients *vs.* CHC patients remains controversial. Given the scarcity of organ sources, surgeons and ethicists need to adjust organ allocation criteria on the basis of future research.

## 5 Conclusion

In conclusion, liver transplantation confers an advantage in OS and CSS for CHC patients. Liver transplantation may be feasible for patients with CHC and should be taken into consideration. However, a sizable sample is required for future studies to determine which subset of CHC patients may benefit more from liver transplantation.

## Funding Sources

This work was supported by grants from the National Key Technologies R&D Program (2018YFC1106800), the 10.13039/501100001809Natural Science Foundation of China (82170,621, 82070644, 81800564 and 81770615), and the 1.3.5 project for disciplines of excellence, 10.13039/501100013365West China Hospital, Sichuan University (ZYJC18008).

## Statement of ethics

Because all patient information in the SEER database has been deidentified and is publicly accessible, the research involving the anonymous data from that database was exempt from medical ethics review and did not require informed consent.

## Data availability statement

The datasets generated during and/or analyzed during the current study are available in the SEER repository.(Surveillance, Epidemiology, and End Results (SEER) Program (www.seer.cancer.gov) SEER*Stat Database: Incidence - SEER Research Data, 18 Registries, Nov 2020 Sub (2000–2018) - Linked To County Attributes - Time Dependent (1990–2018) Income/Rurality, 1969–2019 Counties, National Cancer Institute, DCCPS, Surveillance Research Program, released April 2021, based on the November 2020 submission).

## CRediT authorship contribution statement

**Shizheng Mi:** Software, Visualization, Writing – original draft, Writing – review & editing. **Ziqi Hou:** Formal analysis, Software, Validation. **Guoteng Qiu:** Formal analysis, Investigation. **Zhaoxing Jin:** Conceptualization, Software. **Jiwei Huang:** Conceptualization, Funding acquisition, Methodology.

## Declaration of competing interest

The authors declare that they have no known competing financial interests or personal relationships that could have appeared to influence the work reported in this paper.

## References

[1] GLOBOCAN cancer fact sheets: liver cancers n.d. https://gco.iarc.fr/.

[2] Sung H., Ferlay J., Siegel R.L., Laversanne M., Soerjomataram I., Jemal A. (2021). Global cancer statistics 2020: GLOBOCAN estimates of incidence and mortality worldwide for 36 cancers in 185 countries. Ca - Cancer J. Clin..

[3] Bridgewater J., Galle P.R., Khan S.A., Llovet J.M., Park J.W., Patel T. (2014). Guidelines for the diagnosis and management of intrahepatic cholangiocarcinoma. J. Hepatol..

[4] Llovet J.M., Zucman-Rossi J., Pikarsky E., Sangro B., Schwartz M., Sherman M. (2016). Hepatocellular carcinoma. Nat. Rev. Dis. Prim..

[5] Brunt E., Aishima S., Clavien P.A., Fowler K., Goodman Z., Gores G. (2018). cHCC-CCA: consensus terminology for primary liver carcinomas with both hepatocytic and cholangiocytic differentation. Hepatology.

[6] Ramai D., Ofosu A., Lai J.K., Reddy M., Adler D.G. (2019). Combined hepatocellular cholangiocarcinoma: a population-based retrospective study. Am. J. Gastroenterol..

[7] Jarnagin W.R., Weber S., Tickoo S.K., Koea J.B., Obiekwe S., Fong Y. (2002). Combined hepatocellular and cholangiocarcinoma: demographic, clinical, and prognostic factors. Cancer.

[8] Lee C.C., Wu C.Y., Chen J.T., Chen G.H. (2002). Comparing combined hepatocellular-cholangiocarcinoma and cholangiocarcinoma: a clinicopathological study. Hepato-Gastroenterology.

[9] Allen R.A., Lisa J.R. (1949). Combined liver cell and bile duct carcinoma. Am. J. Pathol..

[10] Goodman Z.D., Ishak K.G., Langloss J.M., Sesterhenn I.A., Rabin L. (1985). Combined hepatocellular-cholangiocarcinoma. A histologic and immunohistochemical study. Cancer.

[11] Bosman F.T., Carneiro F., Hruban R.H., Theise N.D. (2010). Pathology and Genetics of Tumours of the Digestive System.

[12] Nagtegaal I.D., Odze R.D., Klimstra D., Paradis V., Rugge M., Schirmacher P. (2020). The 2019 WHO classification of tumours of the digestive system. Histopathology.

[13] Beaufrère A., Calderaro J., Paradis V. (2021). Combined hepatocellular-cholangiocarcinoma: an update. J. Hepatol..

[14] Wakizaka K., Yokoo H., Kamiyama T., Ohira M., Kato K., Fujii Y. (2019). Clinical and pathological features of combined hepatocellular-cholangiocarcinoma compared with other liver cancers. J. Gastroenterol. Hepatol..

[15] Garancini M., Goffredo P., Pagni F., Romano F., Roman S., Sosa J.A. (2014). Combined hepatocellular-cholangiocarcinoma: a population-level analysis of an uncommon primary liver tumor. Liver transplantation : official publication of the American Association for the Study of Liver Diseases and the. International Liver Transplantation Society.

[16] Magistri P., Tarantino G., Serra V., Guidetti C., Ballarin R., Di Benedetto F. (2017). Liver transplantation and combined hepatocellular-cholangiocarcinoma: feasibility and outcomes. Digestive and liver disease. official journal of the Italian Society of Gastroenterology and the Italian Association for the Study of the Liver.

[17] Surveillance, epidemiology, and end results (SEER) Program. http://www.seer.cancer.gov.

[18] Ho D., Imai K., King G., Stuart E.A. (2011). MatchIt: nonparametric preprocessing for parametric causal inference. J. Stat. Software.

[19] Therneau Terry M., Grambsch P.M. (2000).

[20] Kassambara A., Kosinski M., Biecek P., Fabian S. survminer: R package version 0.4.9. https://CRAN.R-project.org/package=survminer.

[21] Facciuto M.E., Singh M.K., Lubezky N., Selim M.A., Robinson D., Kim-Schluger L. (2015). Tumors with intrahepatic bile duct differentiation in cirrhosis: implications on outcomes after liver transplantation. Transplantation.

[22] Schizas D., Mastoraki A., Routsi E., Papapanou M., Tsapralis D., Vassiliu P. (2020). Combined hepatocellular-cholangiocarcinoma: an update on epidemiology, classification, diagnosis and management. Hepatobiliary & pancreatic diseases international. HBPD INT.

[23] Groeschl R.T., Turaga K.K., Gamblin T.C. (2013). Transplantation versus resection for patients with combined hepatocellular carcinoma-cholangiocarcinoma. J. Surg. Oncol..

[24] Vilchez V., Shah M.B., Daily M.F., Pena L., Tzeng C.W., Davenport D. (2016). Long-term outcome of patients undergoing liver transplantation for mixed hepatocellular carcinoma and cholangiocarcinoma: an analysis of the UNOS database. HPB : the official journal of the International Hepato Pancreato Biliary Association.

[25] Chang C.C., Chen Y.J., Huang T.H., Chen C.H., Kuo F.Y., Eng H.L. (2017). Living donor liver transplantation for combined hepatocellular carcinoma and cholangiocarcinoma: experience of a single center. Ann. Transplant..

[26] Dageforde L.A., Vachharajani N., Tabrizian P., Agopian V., Halazun K., Maynard E. (2021). Multi-center analysis of liver transplantation for combined hepatocellular carcinoma-cholangiocarcinoma liver tumors. J. Am. Coll. Surg..

[27] Lunsford K.E., Court C., Seok Lee Y., Lu D.S., Naini B.V., Harlander-Locke M.P. (2018). Propensity-matched analysis of patients with mixed hepatocellular-cholangiocarcinoma and hepatocellular carcinoma undergoing liver transplantation. Liver transplantation : official publication of the American association for the study of liver diseases and the. International Liver Transplantation Society.

[28] Jaradat D., Bagias G., Lorf T., Tokat Y., Obed A., Oezcelik A. (2021). Liver transplantation for combined hepatocellular-cholangiocarcinoma: outcomes and prognostic factors for mortality. A multicenter analysis. Clin. Transplant..

[29] De Martin E., Rayar M., Golse N., Dupeux M., Gelli M., Gnemmi V. (2020). Analysis of liver resection versus liver transplantation on outcome of small intrahepatic cholangiocarcinoma and combined hepatocellular-cholangiocarcinoma in the setting of cirrhosis. Liver transplantation : official publication of the American association for the study of liver diseases and the. International Liver Transplantation Society.

[30] Wang J., Li Z., Liao Y., Li J., Dong H., Peng H. (2021). Prediction of survival and analysis of prognostic factors for patients with combined hepatocellular carcinoma and cholangiocarcinoma: a population-based study. Front. Oncol..

[31] Chen X., Sun S., Lu Y., Shi X., Wang Z., Chen X. (2022). Promising role of liver transplantation in patients with combined hepatocellular-cholangiocarcinoma: a propensity score matching analysis. Ann. Transl. Med..

